# Effects of acid mine drainage from collapsed tailings dam on Zambia’s human riparian communities: a fuzzy cognitive model perspective

**DOI:** 10.1038/s41598-025-34577-0

**Published:** 2026-01-08

**Authors:** Vincent R. Nyirenda, Lackson Chama, Darius Phiri, Confred G. Musuka, Grant Simuchimba, Moses Chibesa, Matamyo Simwanda, Mazuba Siamujompa, Robby Kasubika, Sydney Kapembwa, Stanford Siachoono, Abbie C. Lwali, Ngawo Namukonde, Wilfred Nyirenda, Nelly Chunda-Mwango, Edwin Kikamba, Chisomo J. Phiri, Bongani M’doma, Bimo A. Nkhata, Benjamin Mubemba, Yuji Murayama

**Affiliations:** 1 Department of Zoology and Aquatic Sciences, School of Natural Resources, The Copperbelt University, P.O. Box 21692, Kitwe, Zambia; 2 Department of Plant and Environmental Sciences, School of Natural Resources, The Copperbelt University, P.O. Box 21692, Kitwe, Zambia; 3 Department of Clinical Sciences, School of Medicine, The Copperbelt University, P.O. Box 21692, Ndola, Zambia; 4 African Centre of Excellence for Sustainable Mining, The Copperbelt University, P.O. Box 21692, Kitwe, Zambia; 5 Department of Fisheries, National Aquaculture Research and Development Centre, P.O. Box 22797, Kitwe, Zambia; 6 Centre for Environmental Management, Faculty of Natural and Agricultural Sciences, University of The Free State, P.O. Box 339, Bloemfontein, 9300 Republic of South Africa; 7 Faculty of Life and Environmental Sciences, University of Tsukuba, 1-1-1 Tennodai, Tsukuba City, 305-8572 Ibaraki Japan

**Keywords:** Acid mine drainage, Environmental disaster, Emergency planning, Risk analysis, Social-ecological system, Sustainable development goals, Ecology, Environmental sciences, Environmental social sciences, Diseases, Health care, Risk factors, Engineering

## Abstract

Acid Mine Drainage (AMD) is a major global environmental and public health concern, particularly in mining areas. Following the collapse of the tailings dam at Sino Metals Leach Limited Mine in Zambia’s Copperbelt Province and the subsequent discharge of mining effluents into the Mwambashi River, a cross-sectional study was conducted to assess the impacts on adjoining human riparian communities. Using the Mentor Modeler software for Fuzzy Cognitive Mapping (FCM), we analysed and predicted the socio-ecological effects of AMD on the communities living adjacent to riparian areas. The study revealed that AMD and the consequent decline in water quality triggered a cascade of adverse socio-economic and health impacts among the communities living adjacent to riparian areas. Understanding the FCMs in emergencies related to such impacts is crucial for targeted interventions, particularly in data-limited environments. These insights can inform more effective allocation of limited financial and technical resources, enhance decision-making by the authorities, and support the development of community-based strategies to empower affected communities and mitigate or prevent future AMD-related disasters.

**Supplementary Information:**

The online version contains supplementary material available at 10.1038/s41598-025-34577-0.

## Introduction

Mining activities are a major driver of economic growth in Zambia^1^. However, mining-related ecotoxicological risks, particularly from acidic conditions and heavy metals, pose serious public health concerns and have far-reaching socio-economic and environmental consequences^2–5^. Besides, slag material exposure to acidic environments, and increased slag weathering promote release of heavy metals, such as zinc, lead and nickel into adjacent environment^6^. Tailings dam failure can occur due to various factors, such as overtopping, earthquakes, weak foundations, and liquefaction^7^. When tailings containing sulphide minerals are exposed to air and water, they oxidise and generate Acid Mine Drainage (AMD) – a highly acidic effluent enriched with toxic heavy metals, such as copper, cobalt, lead, cadmium, arsenic, and chromium^8,9^. These pollutants severely degrade water quality, leading to biodiversity loss and health risks to wildlife, livestock and humans who depend on contaminated water bodies^10^. The direct effects of AMD include a severe lowering of pH (< 4.0), which is significantly below the normal pH range of 6.5 to 8.5^11^, and the ability to mobilise toxic metals into environmental contaminants in soils and water systems^12^. Consequently, these pollutants impact livelihoods, human health and agricultural productivity.

While several studies have examined the environmental and socio-economic consequences of mining, particularly concerning AMD, there remains a critical gap in how these impacts are systematically mapped and modelled in data-limited contexts, such as sub-Saharan Africa^2,4^. Traditional modelling approaches often depend on deterministic or statistical frameworks that may lack the flexibility to incorporate local knowledge, real-time community feedback, and non-linear system dynamics^12,13^. These approaches tend to overlook the subjective and systemic dimensions of environmental risk that are vital for effective emergency response and policy development.

This study focused on the recent collapse of the tailings dam at Sino Metals Leach Zambia Limited, a Chinese-owned copper mine, reported on 18^th^ February 2025. The incident resulted in the discharge of approximately 50 million litres of acidic mine waste into the Mwambashi and Kafue Rivers in Zambia’s Copperbelt Province, leading to water quality degradation, soil contamination, biodiversity loss, and socio-economic disruptions over extended temporal and spatial scales. The acidic effluent caused mass mortality of fish and other aquatic organisms, severely impacting food security and economic activities. Human communities residing along these river systems lost livelihoods; fishermen and traders abandoned their fish enterprises; livestock, such as cattle, goats and poultry died; and crops were also affected. As a result, affected human communities suffered from a range of health issues, including skin and diarrhoeal diseases^14^.

To assess and predict the complex effects of AMD from the collapsed tailings dams on riparian communities, the study employed a Fuzzy Cognitive Mapping (FCM) approach. The FMC is a semi-quantitative modelling technique that combines expert knowledge with stakeholder perceptions to analyse causal relationships among environmental and socio-economic variables^15^. Unlike traditional deterministic models, FCM accommodates uncertainty and non-linearity in system interactions, making it particularly suitable for addressing complex environmental challenges, such as AMD pollution^16^. To date, few studies have employed participatory modelling tools, such as FCM, to integrate community perspectives with expert input, especially in the context of AMD in African mining regions^17,18^. The FCM is particularly valuable in data-scarce, low-data, emergency or rapidly changing contexts because it depends on expert knowledge rather than extensive empirical datasets, enabling rapid conceptualization of system dynamics when time or data are a constraint. Its qualitative-quantitative structure allows quick updates, scenario testing, and decision support under uncertainty, making it highly adaptive during fast-moving or data-scarce crises. By structuring relationships between variables as weighted connections, FCM enables researchers and participants to co-produce cognitive networks with respondents, simulate different scenarios, and assess the potential consequences of environmental disasters. It also helps identify potential mitigation strategies or intervention points. Rooted in graph theory^19^, FCM offers a flexible, participatory decision-support tool that is increasingly recognised for its applicability to complex environmental decision-making^20,21^. When used effectively, it can contribute to the development of long-term, sustainable mitigation strategies^22^.

Although there has been an increase in the application of FCMs in environmental modelling, their use in AMD studies has remained limited to static classification or conceptual mapping, with very few attempts to integrate empirical stakeholder socio-hydro-ecological perspectives into dynamic, uncertainty-aware causal simulations. Recent methodological advances in FCMs, such as probabilistic weighting and hybrid optimisation linkages have not yet been largely operationalised for AMD risk assessment and mitigation planning. This study addresses that gap by developing a hybrid, field-validated FCM that combines stakeholder-elicited causal structure with propagation of uncertainty in influence weights. In doing so, it provides one of the first empirically grounded, participatory FCM frameworks capable of simulating AMD system trajectories and evaluating realistic mitigation scenarios.

Guided by the Theory of Change (ToC), the study postulated that if remedial strategies are implemented according to the pre-set goals, positive outcomes are likely to be realised^23^. Additionally, the research was conceptualised within the Drivers-Activities-Pressure-State changes-Impacts on Welfare Response (as Measures) (DAPSI(W)R(M) framework^24^. The framework posits that AMD resulting from the mining activities degrades the environment, undermines ecosystem integrity, and diminishes both ecosystem health and human well-being, thereby necessitating targeted intervention strategies. The main objective of the study was to assess and predict the socio-economic and environmental impacts of AMD on livelihoods, ecosystem and human health, and agricultural productivity, as well as evaluate associated remedial strategies. The research outcomes have strong potential to support the achievement of several Sustainable Development Goals (SDGs), including SDG 1 [No poverty], SDG 2 [Zero hunger], SDG 6 [Clean water and sanitation], SDG 9 [Industrial, innovation and infrastructure], SDG 14 [Life below water], and SDG 15 [Life on land]. The study is guided by the following research questions: (i) What are the causal linkages in the FCM model associated with AMD affecting riparian communities? (ii) Which components in the AMD network are most influential? and (iii) How do system components respond to changes in environmental state or magnitude? The findings are intended to inform policymakers, conservationists, practitioners, academia, and local communities by providing a decision-support tool for developing effective disaster response strategies. Ultimately, the model supports the creation of an early warning system, and promotes integrated approaches to biodiversity conservation and sustainable livelihood strategies.

## Methods and materials

### Study area

**Fig. 1:**
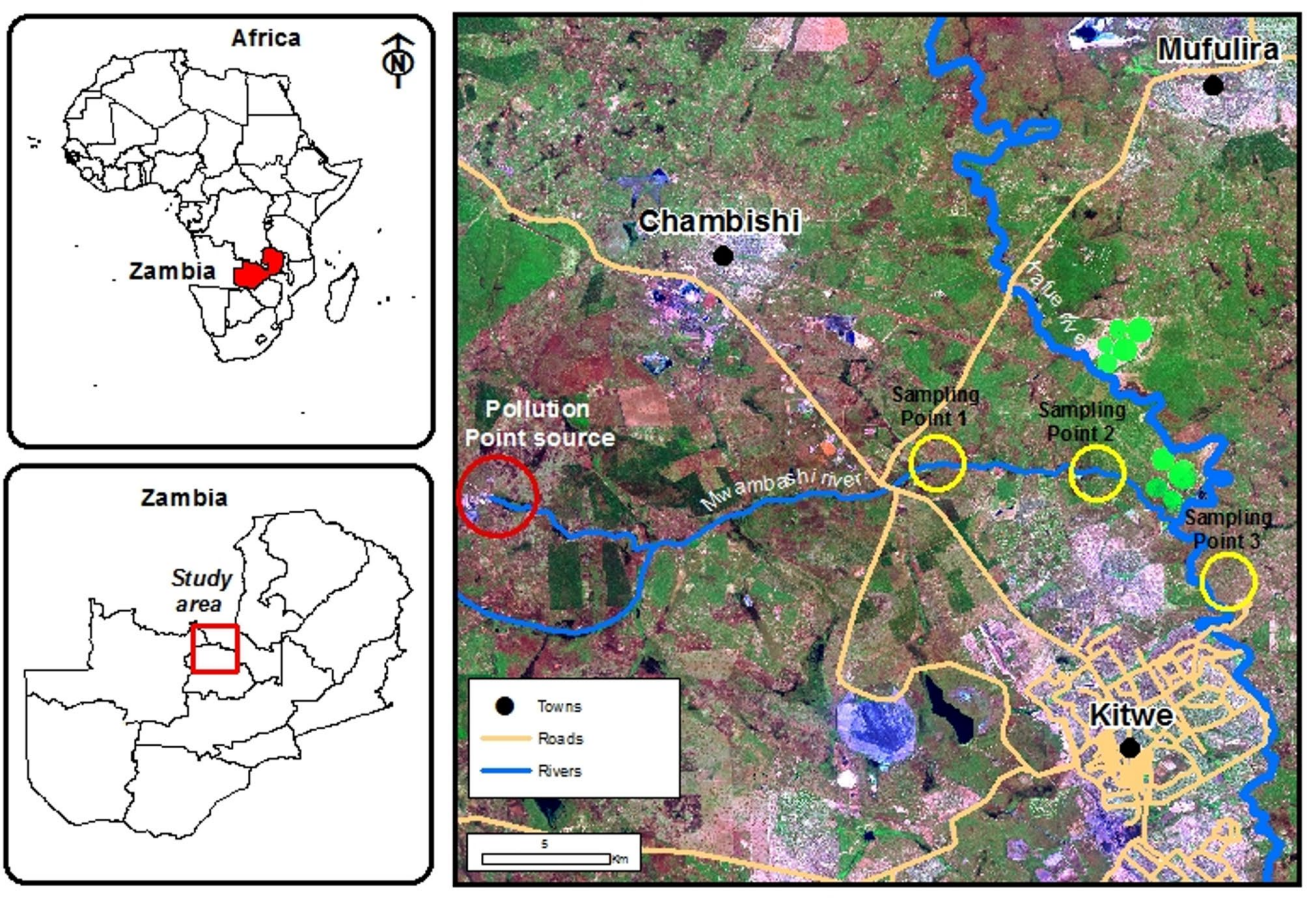
Sampling points for the acid mine drainage pollution along the Mwambashi and Kafue Rivers in Kitwe, 2025. Figure 1 was generated using ArcMap 10.7.1 (Esri, Redlands, California) and multiple datasets, including a shapefile of Zambian rivers, a road layer, and Landsat 9 satellite imagery used as a basemap.

The study was conducted in AMD-affected areas, flanking the Mwambashi and Kafue Rivers in Zambia’s Copperbelt province – a region historically dominated by copper mining activities (Fig. 1). The area has long been exposed to mine-derived heavy metals, contaminating environment, including soils, rivers and groundwater^25^. With the recent expansions of mining exploration and operations across Zambia^26^, the area has experienced an increase in sporadic AMD incidents originating from multiple mining sites^2^.

The livelihoods of residents in the study area largely depend on subsistence and small-scale commercial agriculture, with common crops including maize (*Zea mays*), carrots (*Daucus carota*), okra (*Abelmoschus esculentus*), rape vegetables (*Brassica napus*), tomatoes (*Solanum lycopersicum*), cabbages (*Brassica oleracea*) and eggplant (*Solanum melongena*). The local economy also relies heavily on the riparian ecosystem, supporting a range of activities, such as fishing, livestock rearing, fish farming, and commodity trading. Key fish species in the Mwambashi and Kafue Rivers include cichlids; greenhead tilapia (*Oreochromis macrochir*), three-spotted tilapia (*O. andersonii)* and Nile tilapia (*O. niloticus)*; red-breasted tilapia (*Coptodon rendalli*), banded tilapia (*Tilapia* sparrmanii) and African catfish (*Clarias gariepinus*). The rivers also provide non-fish resources, such as reeds (*Phragmites australis*) for mat-making, and sedges (*Carex spp*.) for basketry. Livestock farmers commonly rear cattle, goats, sheep and poultry animals, such as chickens and ducks. These entrepreneurs find markets for field-based and aquatic products in nearby mining towns of Kitwe, Mufulira and Kalulushi, which have human populations of 661 901, 200 182 and 170 701, respectively^27^, as well as in border town of Kasumbalesa in the Democratic Republic of the Congo.

The study area experiences three distinct climatic seasons: hot-wet, cold-dry, and hot-dry, and lies within the Agro-Ecological Region III, characterised by annual rainfall ranging from 1, 000 to 1, 200 mm and mean annual temperatures ranging from 12 ˚C to 29 ˚C^28^. The area’s geology is rich in copper-bearing deposits^29^ and features flat topography drained by the Mwambashi and Kafue Rivers. These physio-geological conditions are likely to influence both the occurrence and spatial spread of AMD and its associated ecotoxicological impacts.

### Study population and data collection procedures

The data collection was conducted under the ethical oversight of The Copperbelt University. All data collection methods were performed in accordance with 2024 Helsinki declaration regarding human participants. For instance, free prior informed consent was obtained from participants, confidentiality of volunteered information was assured and kept, while ensuring avoidance of inflicting harm on them as a result of the study. Prior to data collection, a semi-structured questionnaire (Supplementary Information - Appendix A.1) consisting of closed and open questions was developed and pre-tested for clarity and validity by five independent residents from the study area. Experienced and well-trained enumerators, fluent in both English and Bemba – the vernacular language collected the data during the survey that lasted about 40 min per respondent. All the respondents demonstrated proficiency in English, Bemba or both. Data were collected and digitally recorded using *Kobo Collect* – an open-source application designed for robust field-based data collection.

To minimise potential bias related to emotional distress or expectations of compensation, the survey was conducted in March 2025, approximately one month after the AMD incident. At the time, compensation-related discussions were ongoing, primarily centred on the loss of livelihoods, health, and agricultural productivity. Concurrently, stakeholders, including Public Health staff from Zambia’s Ministry of Health, were engaged in community outreach to raise awareness about environmental and health risks.

The study employed purposive sampling to identify respondents directly affected by the AMD incident, following protocols recommended for unknown population sizes in emergency settings^30,31^. The sampling began with a key informant interview, which helped identify “information-rich” individuals knowledgeable about the event and its impact. Specific criteria used for respondent selection (adapted from^32^, included (i) awareness of the AMD incident; (ii) a resident of the affected area; (iii) affected by the AMD in one way or another; and (iv) willingness to participate in the survey and share relevant information. From the initial respondent, a snowball sampling technique was employed to interview respondents individually and physically face-to-face, where the next respondent was identified through peer referencing by the previous respondent^33^. The cut-off point for the sample size was reached when saturation was achieved with the 18^th^ respondent, in line with previous studies that have established that 9–17 interviews suffice to attain saturation^34^. This sample size is also consistent with the requirements of FCM approach, which does not need a large or specific sample size.

### Model development

Shared mental models (i.e., Fuzzy Cognitive Maps, FCM) were developed based on the collated data provided by respondents. Therefore, the interpersonal disparities in the availed information were averaged. Detailed explanation on how the averaging can be achieved is provided by^35^. The resultant mental maps consisted of components or elements that were linked by weighted perceived causal values as perceived by the respondents. The rating was drawn from a 5-Likert scale, ranging from 1 to 5, representing lowest to highest, which provided the magnitude of the effects of the AMD experienced. Furthermore, the questions in the questionnaire provided respondents with possible dimensional directions (positive or negative) of the AMD effects (Appendix A.1), which required confirmation or counter-confirmation. The rating was then transformed on a -1 to + 1 scale in the mapping environment by multiplying the acquired values by 2.

For every causal link identified by respondents, individual influence weights were first standardised from questionnaire-based Likert scale ratings to a common fuzzy scale (–1 to + 1). We aggregated weights provided by multiple participants for the same link using the arithmetic mean. Prior to aggregation, all respondent matrices were normalised using min–max rescaling to reduce inter-individual variability in scale use, and links were retained only if at least ≥ 30% respondents indicated its presence and if the mean absolute weight exceeded a minimum threshold − 1.0. Consistency checks included screening for outlier weights, and verifying that the aggregated adjacency matrix remained structurally coherent (no contradictory reciprocal links with opposite signs).

The development of the FCM model was implemented in four sequential steps: (i) establishing causal relationships between system components/elements bearing dimensions and magnitudes; (ii) constructing adjacency matrices to represent the relationships between paired elements (dyads); (iii) model validation using key network structural metrics; and (iv) conducting scenario analysis to explore potential outcomes under varying intervention strategies. The resultant model represented integrated network structural properties (indegrees, outdegrees, and centrality), where indegree represents inward associations, outdegree the outward associations, and centrality represents the significance of components or elements of AMD. Furthermore, model validation also consisted of these network structural properties. The adjacency matrices linked dyads of elements with dimensions (positive or negative) and magnitudes (weighted) in the model.

To co-produce predictable AMD mitigations and strategies that reflect patterns of planned outcomes, scenario analyses were conducted. The construction of the scenarios for preferred outcomes was based on considering the drivers, ordinaries and receivers in the models. Drivers refer to influencer components that have considerable effects on the other components in the model. Ordinaries are regular components contributing domino effects to the overall model, while receivers refer to components influenced by other components.

Model robustness was evaluated and supported through comparison of FCM-predicted system responses with evaluations from independent domain experts, who did not participate in the original elicitations, but confirmed that the model’s projected directions and relative magnitudes of change aligned with established AMD pollution behaviour and socio-ecological effects. The convergence between expert expectations and model outputs strengthening confidence in the predictive credibility and reliability of the final consensus FCM for management decision-making.

### Data analysis

To visualise and interpret the perceived effects of AMD, data were analysed using Mental Modeler (https://www.mentalmodeler.com) – an open-access participatory software specifically designed for constructing and simulating FCM. As noted by^18^, Mental Modeler is among several platforms that facilitate the co-construction of FCMs to integrate local and expert knowledge in complex decision-making contexts. These models characterise collective intelligence as input into the co-production of change analysis, state preferences and solutions in social-ecological systems^17^. The scenario analysis identified anticipated system performance under different settings, with positive or negative feedbacks^36^. Using the Mental Modeler’s Scenario module, three scenarios were created based on the respondents’ views regarding the shared mental models of AMD. Scenario 1: reducing AMD levels, which focused on minimizing pollution at the source; Scenario 2: Mitigating the effects of driver components, targeting highly influential variables that shape system behaviour; and Scenario 3: reducing the impacts of ordinary components, which aimed at interrupting cascading intermediate effects within the system.

## Results

### Demographic characteristics

Data were collected from both male (16) and female (2) respondents, aged between 31 and 65 years old, all of whom were married, with family sizes ranged from 3 to 13 (median = 6). The majority of respondents were predominantly from Bemba ethnicity, while the rest were from other ethnic groupings, such as Chewa, Lamba, Lunda, Mambwe, Nsenga, Shona and Tumbuka. In terms of education, respondents attained primary (*n* = 7), secondary (*n* = 9) and tertiary (*n* = 2). The respondents had diverse primary occupations *inter alia*, bricklayer (*n* = 1), dairy farmer (*n* = 1), driver (*n* = 1), farmer (*n* = 13), watchman (*n* = 1), fishermen (*n* = 3) and traders (*n* = 3), combined with various other multiple secondary occupations.

### Network measures

**Fig. 2:**
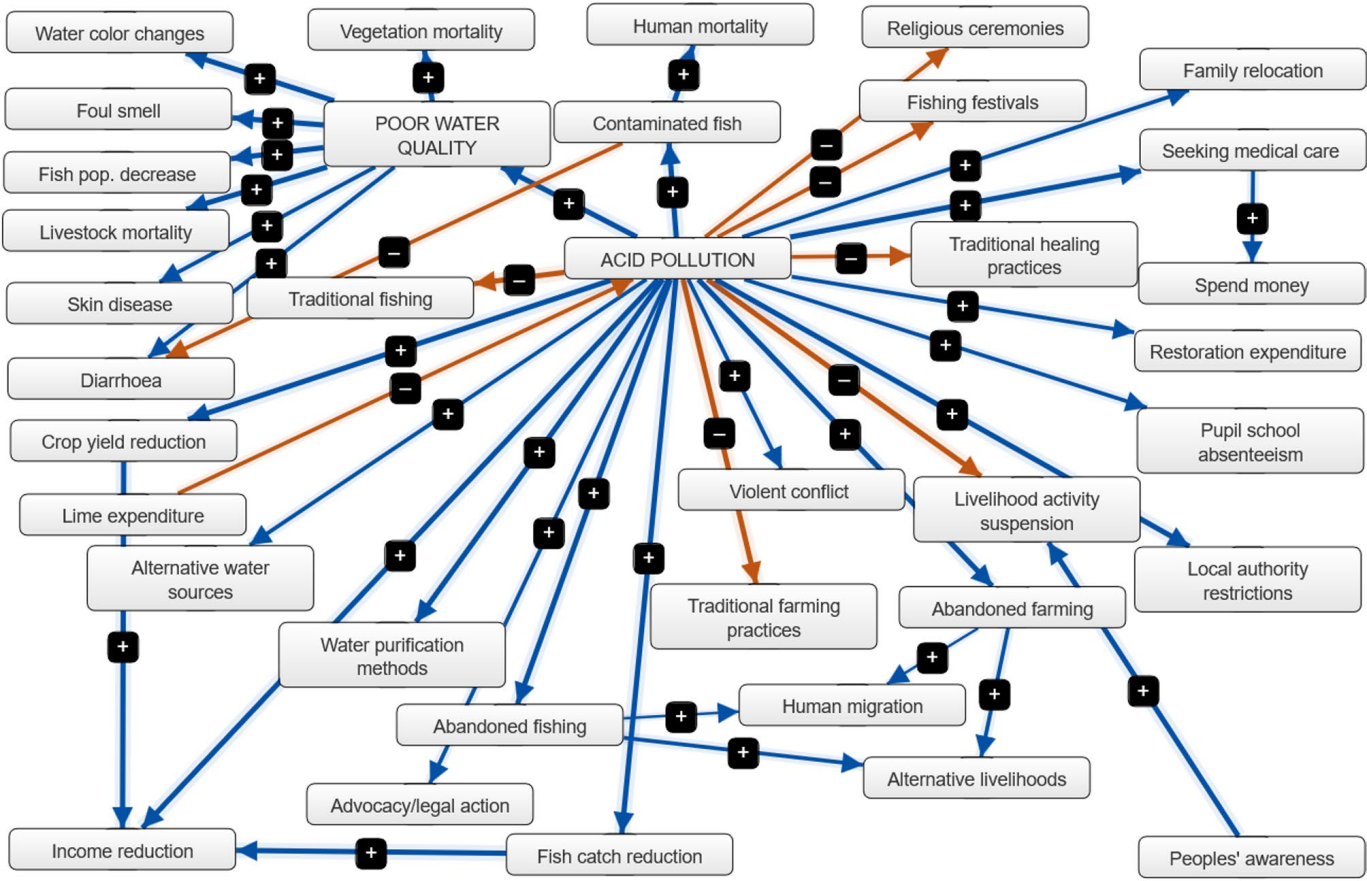
Components and their relationships relating to acid mine drainage pollution affecting the riparian communities along Zambia’s Mwambashi and Kafue Rivers in Kitwe, Zambia,2025. The blue arrows represent the positive causal effects between the components, while the brown arrows depict the negative causal effects between the components. The weighted thickness of the lines reflects the strength of the relationships, while the arrowheads indicate the direction of the effects.

The network structure for the AMD as presented in Fig. 2, comprises its constituencies (Table 1). As an index of its complexity, with several possible outcomes and implications, the number of the receiver (R) components was higher than drivers (T) and ordinary components combined. There were few and well-elaborated system-based transmitters or drivers (2), resulting in a large R/T ratio of 13 for a complex cognitive map (Table 1).

**Table 1:** Network measures of the fuzzy cognitive map for acid mine drainage pollution along Mwambashi and Kafue Rivers in Kitwe, Zambia, 2025, together with associated references.

Measures	Total	References
Components (elements)	36	^37,38^
Total connections	40	
Density	0.032	
Connections per component	1.111	
Number of driver components	2	
Number of receiver components	26	
Number of ordinary components	8	
Complexity score	13	

### Model relational linkages

In the FCM, each relationship had a strength greater than + 0.5 or less than − 0.5, except for those relating to human migration, as only one case of migration was recorded during the survey. The most central, important, and influential variable in the FCM was AMD, followed by the poor water quality (Fig. 2, Figures SI 1–2; Table 2). Both of these variables, as ordinaries in the FCM, contributed to the overall model through domino effects (Table 2). Other issues (ordinaries) included contaminated fish, abandoned fishing and farming activities, reduced fish catch and crop yields, and seeking medical care. The total number and species of fish, crops, and other vegetation lost to AMD in the affected area were unknown, but considered huge by the respondents and stakeholders. In the case of income lost by respondents as a result of AMD within one month of occurrence, the range was from ZMW 200 to ZMW 70, 000, with a median of ZMW 15, 000 per household (US$1: ZMW28.63). In addition, the medical costs incurred by respondents ranged from ZMW 100 to ZMW 25, 000, with a median of ZMW 170 per household. People’s awareness and lime expenditure were the drivers with considerable effects in this FCM, while the remaining variables (26) in the model were recipients (receivers) that were affected by other variables and highlighted the implications of the AMD (Table 2).

**Table 2:** Adjacency matrix for indegrees, outdegrees and centrality of acid mine drainage pollution along the Mwambashi and Kafue Rivers in Kitwe, Zambia, 2025.

Component	Indegree	Outdegree	Centrality	Type
Acid pollution	0.80	19.04	19.84	Ordinary
Poor water quality	0.96	6.30	7.26	Ordinary
Income reduction	2.76	0.00	2.76	Receiver
Contaminated fish	0.98	1.74	2.72	Ordinary
Abandoned fishing	1.00	1.08	2.06	Ordinary
Abandoned farming	1.00	1.08	2.08	Ordinary
Livelihood activity suspension	1.92	0.00	1.92	Receiver
Fish catch reduction	0.98	0.92	1.90	Ordinary
Crop yield reduction	0.98	0.92	1.90	Ordinary
Seeking medical care	0.98	0.70	1.68	Ordinary
Alternative livelihoods	1.60	0.00	1.60	Receiver
Diarrhoea	1.48	0.00	1.48	Receiver
Water purification methods	1.00	0.00	1.00	Receiver
Human mortality	1.00	0.00	1.00	Receiver
Vegetation mortality	0.98	0.00	0.98	Receiver
Livestock mortality	0.96	0.00	0.96	Receiver
Fish population decrease	0.96	0.00	0.96	Receiver
Foul smell	0.96	0.00	0.96	Receiver
Water colour changes	0.96	0.00	0.96	Receiver
People’s awareness	0.00	0.92	0.92	Driver
Traditional farming practices	0.92	0.00	0.92	Receiver
Traditional fishing	0.92	0.00	0.92	Receiver
Local authority restrictions	0.84	0.00	0.84	Receiver
Alternative water sources	0.80	0.00	0.80	Receiver
Lime expenditure	0.00	0.80	0.80	Driver
Family relocation	0.76	0.00	0.76	Receiver
Violet conflict	0.74	0.00	0.74	Receiver
Pupil school absenteeism	0.74	0.00	0.74	Receiver
Skin diseases	0.74	0.00	0.74	Receiver
Traditional healing practices	0.72	0.00	0.72	Receiver
Fishing festivals	0.72	0.00	0.72	Receiver
Religious ceremonies	0.72	0.00	0.72	Receiver
Spend money	0.70	0.00	0.70	Receiver
Advocacy/legal action	0.68	0.00	0.68	Receiver
Restoration expenditure	0.68	0.00	0.68	Receiver
Human migration	0.56	0.00	0.56	Receiver

### Scenario analysis

**Fig. 3:**
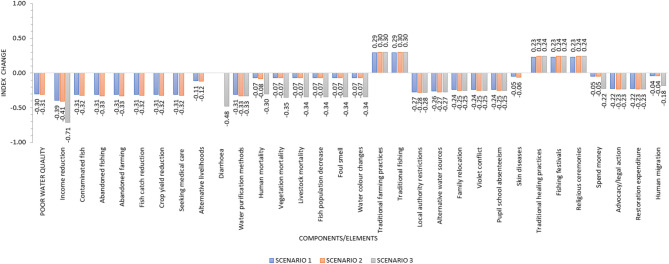
Outcome of scenario analysis for scenarios 1–3 for acid mine drainage pollution along the Mwambashi and Kafue Rivers in Kitwe, Zambia, 2025. Scenario 1 represents reduction of acid mine drainage pollution levels, Scenario 2 additively involves increasing public awareness and reducing lime expenditure, and Scenario 3 additively reducing the effects of ordinaries (i.e., poor water quality, contaminated fish, abandoned fishing, abandoned farming, fish catch reduction, crop yield reduction, and seeking medical care) associated with acid mine drainage pollution along the Mwambashi and Kafue Rivers in Kitwe, Zambia, 2025. Values above zero (+, without a sign) depict positive changes, while those below (-) show negative changes. Thus, the longer the bars, the more the expected change and vice-versa.

To test model sensitivity, three scenario analyses resulted in the outcomes shown in Fig. 3, based on the data presented in Table 2. In Scenario 1, the reduction of AMD had a positive and remarkable direct effect on system components (Fig. 3). However, this scenario did not influence or show that it would likely reduce livelihood activity suspension, people’s awareness or lime expenditure. As such, these components were automatically removed from the model by the algorithm. In Scenario 2, increasing people’s awareness additively and reducing lime expenditure through strategies targeting these drivers resulted in improved overall variables (Fig. 3). Reducing the effects of multiple ordinaries (i.e., poor water quality, contaminated fish, abandoned fishing, abandoned farming, reduced fish catch, reduced crop yield, and increased medical care) in Scenario 3 further improved the AMD situation for the residents (Fig. 3).

In relation to Fig. 3, the AMD initiates a chain of negative impacts that propagate through both ecological and socio-economic systems. The deterioration of water quality through acidification and heavy-metal contamination directly undermines fishing and farming, reducing yields and eroding household income. Poor water quality also compromises human and animal health, and in more severe cases contributing to mortality. As traditional livelihoods become less viable, people increasingly turn to alternative livelihoods, while others face relocation pressures. Local authorities impose restrictions on water use, land access, or settlement patterns, intensifying socio-economic stress. Scarcer resources and shifting livelihoods heighten conflicts within communities and between multiple stakeholders. These AMD-induced pressures disrupt education, as children leave school when affected, and to support household income or relocate with families. AMD also undermines social and cultural practices tied to clean water bodies and land, weakening community cohesion. In response, affected populations mobilise through advocacy or legal action, demanding accountability and remediation. Over the long term, efforts toward environmental restoration become essential in AMD affected areas to rebuild ecosystem functions and re-establish stable, sustainable livelihoods.

## Discussion

### Complexity and system vulnerability

Applying FCM to characterise the network structure of AMD along the Mwambashi and Kafue River in Zambia, revealed a highly complex system, primarily driven by external influences rather than internally generated dynamics. The respondent-driven components in the network exhibited strong causal linkages, underscoring the interconnectedness and systemic vulnerability of riparian communities to AMD impacts. This configuration suggests that these communities are highly exposed to environmental shocks, with limited internal buffering capacity. The findings align with previous environmental studies that have applied FCM, where the dominance of receivers in complex systems depicts high socio-ecological stresses and environmental-based impact uncertainties (e.g^39,40^. These findings advance understanding of AMD socio-ecological resilience by revealing that the community well-being, livelihoods, and environmental conditions are closely interlinked, amplifying vulnerability rather than absorbing disturbance, and stressing lack of internal buffering mechanisms. Thus, the communities are highly sensitive to even modest environmental shocks from AMD. From policy standpoint, this implies that effective interventions must move beyond single-sector based solutions, such as a water treatment alone, and instead support integrated resilience-building strategies, including livelihood diversification, strengthened local governance, early-warning and monitoring systems, and targeted social protection. Such evidence helps policy-makers prioritise investments that reduce systemic vulnerability and enhance the community’s capacity to adapt, recover, and sustain ecosystem functions under continued AMD pressure.

### Key drivers and impacts

The AMD and poor water quality emerged as the most central and influential ordinary variables within the FCM network. Their centrality signifies their unique role in triggering a chain of downstream impacts, such as fish contamination, abandonment of fishing and farming activities, reduced fish catches and crop yields, and increased healthcare costs. These findings are consistent with previous research that underscores the cascading effects of water pollution on livelihoods and public health in resource-dependent communities^41^. A comparable incident occurred in 2015 with the collapse of the Fundão tailings dam in Brazil, which discharged approximately 35 million cubic metres of mining waste into the Doce River, and caused severe pollution and lasting environmental degradation^42^. Similarly, the catastrophic failure of the Jagersfontein tailings dam in South Africa released more than 6 million cubic metres of liquid sludge that negatively affected nearly 200 nearby households and approximately 1, 600 ha of agricultural and grazing land; two people died and an additional person remains missing^43^. The prominence of AMD and poor water quality as central variables shows that they are key elements shaping system behaviour, revealing how water degradation triggers wide-ranging socio-ecological vulnerability. Their centrality highlights water quality improvement and AMD mitigation as the most effective policy leverage points for strengthening overall socio-ecological resilience.

The high strength of causal relationships observed in the present study further validates the robustness of the FCM model, except for human migration, which appeared as a singular, weakly linked occurrence during the survey. Despite the challenges, the residents demonstrated notable resilience by adapting and coping with the environmental changes. The residents’ demonstrated ability to adapt and cope with environmental changes highlights existing socio-ecological resilience capacities that help buffer communities against AMD-related disturbances. These adaptive behaviours suggest that policies should strengthen and scale locally grounded coping strategies, such as livelihood diversification, community monitoring, and institutional support to enhance long-term resilience to AMD impacts.

Only two driver variables—public awareness and expenditure on lime—were identified, emphasising a limited strategic capacity for proactive management within the system. Several remedial strategies, such as strengthening legal measures, improving tailings dam infrastructure, enhancing environmental monitoring and disaster preparedness, compensating affected communities, and adopting alternative waste disposal methods that bypass river systems, have been discussed and proposed by the respondents to address structural governance and awareness constraints. The implication for a few active drivers in this model is that responses within the system are likely reactive rather than strategic, making effective targeted strategies more critical for positive system behavioural change^44,45^. Treatment technologies for AMD^8,9^, may serve as primary strategic and proactive measures.

### Scenario-based insights

The scenario analysis highlighted distinct policy and management implications. Scenario 1, which isolated the reduction of AMD, led to improvements across several receiver variables. However, it had minimal impact on key components, such as livelihood activities, public awareness, or lime expenditure, suggesting that endpoint pollution control alone may be insufficient for building systemic resilience. Conversely, Scenario 2, which targeted the driver variables, such as awareness and lime expenditure, demonstrated broader systemic improvements. This scenario underscores the need for effective preparedness prior to the occurrence of AMD and the attendant behavioural change among riparian communities living near functional mines. Scenario 3 focused on mitigating the effects of multiple ordinary variables, resulting in improved conditions for these riparian communities, further reinforcing the importance of multi-dimensional strategies that go beyond reactive measures and instead build adaptive capacity within the system.

### Policy and management implications

This study reveals that reducing AMD alone is not a sufficient policy response. Effective mitigation requires a layered approach involving both structural (such as dam engineering and AMD treatment) and soft interventions (including education, community engagement, and compensation frameworks). There is a strong policy case for strengthening the Zambia Environmental Management Agency (ZEMA)’s monitoring and compliance enforcement on tailings management. Additionally, establishing community-based environmental surveillance systems can facilitate early detection and response to pollution events. Integrating AMD preparedness into district-level disaster management plans would help build local institutional resilience. Furthermore, financing lime distribution programs for emergency household use is a practical short-term response to neutralise acidic effects. Finally, creating AMD early warning and rapid response mechanisms would enhance preparedness and minimise damage. These recommendations are particularly relevant for the Ministry of Mines and Minerals Development, the Ministry of Health, and other actors overseeing mining and water resources.

### Comparison with other cases

The systemic vulnerabilities and cascading socio-economic impacts identified in this study are consistent with patterns observed in other global AMD disasters. Notably, the Fundão dam disaster in Brazil in 2015, which released approximately 35 million cubic metres of mining waste into the Doce River, caused severe water contamination, biodiversity loss, and long-term socio-economic disruption^42^. Similar disasters in China, Indonesia, Peru and South Africa have demonstrated how uncontained AMD from mine tailings can lead to prolonged environmental degradation, food insecurity, and social conflict due to inadequate remediation frameworks and weak enforcement of environmental regulations^5,10^. These cases often exhibit a delayed institutional response, challenges in compensating affected communities, and a lack of community preparedness. The parallels across these global events and the findings of this study validate the FCM-based analysis and underscore the urgent need for proactive, participatory strategies to manage AMD risks^17,18^.

### Study limitations

Although this study has several methodological limitations, it retains considerable analytical strength and practical relevance. First, the AMD occurrence coincided with the rainy (hot-wet) season, which may have had a unique influence on observed impacts compared to the cold-dry or hot-dry seasons. While this temporal limitation affects the generalisation of seasonality, it offers valuable real-time insights into peak contamination periods, which are often the most critical for emergency response planning.

Second, the study did not explicitly account for mediating variables, such as the socio-economic status of respondents, which may influence perceptions and coping capacities. However, the purposive sampling and snowballing strategy ensured that only information-rich individuals with firsthand experience of AMD were included, thus capturing the core dynamics of the impact.

Third, although FCM models are semi-quantitative and rely heavily on perceptions and expert judgement, which may introduce bias, they are widely recognised for capturing complexity in data-scarce or emergency situations^15,18^. The triangulation of stakeholder input, the systematic construction of causal relationships, and the scenario-based analysis all contribute to the study’s robustness, despite limitations in statistical precision.

In sum, while numerical generalisability is constrained, the insights generated provide a strong conceptual and strategic foundation for understanding the socio-ecological consequences of AMD and guiding disaster preparedness. Future research can build on these findings by incorporating longitudinal and spatial analyses to further validate community responses and system dynamics over time.

## Conclusion

This study used shared mental models through FCM to examine the socio-ecological impacts of AMD pollution, revealing a complex, externally influenced system marked by significant vulnerabilities across riparian communities. AMD-driven water quality deterioration produces cascading socio-economic and health impacts on riparian communities, underscoring the need for targeted AMD control policies supported by multi-dimensional resilience strategies. While pollution control offers only partial relief, our findings show that durable mitigation requires a blend of technical measures, such as improved tailings dam management and consistent environmental monitoring, and behavioural and institutional interventions that strengthen community awareness, collaboration, and response capacity. These combined approaches would reduce long-term remediation costs and yield more scalable, cost-effective improvements in both environmental conditions and livelihoods. Overall, the results highlight the necessity of integrated, participatory governance frameworks to anticipate and manage the systemic risks associated with AMD.

## Supplementary Information

Below is the link to the electronic supplementary material.

Supplementary Material 1

## Data Availability

The data presented in this study are available on request from the corresponding author due to requested privacy by respondents.
