# Supplementary material for: Effects of acid mine drainage from collapsed tailings dam on Zambia’s human riparian communities: a fuzzy cognitive model perspective
Source: Sci Rep. 2026 Jan 8;16:4618. doi: 10.1038/s41598-025-34577-0 (PMC12868745; doi:10.1038/s41598-025-34577-0)
Supplement: Supplementary file 1 — Supplementary Material 1 [file 41598_2025_34577_MOESM1_ESM.docx]

**Effects of Acid Mine Drainage from Collapsed Tailings Dam on Zambia’s Human Riparian Communities: A Fuzzy Cognitive Model Perspective**


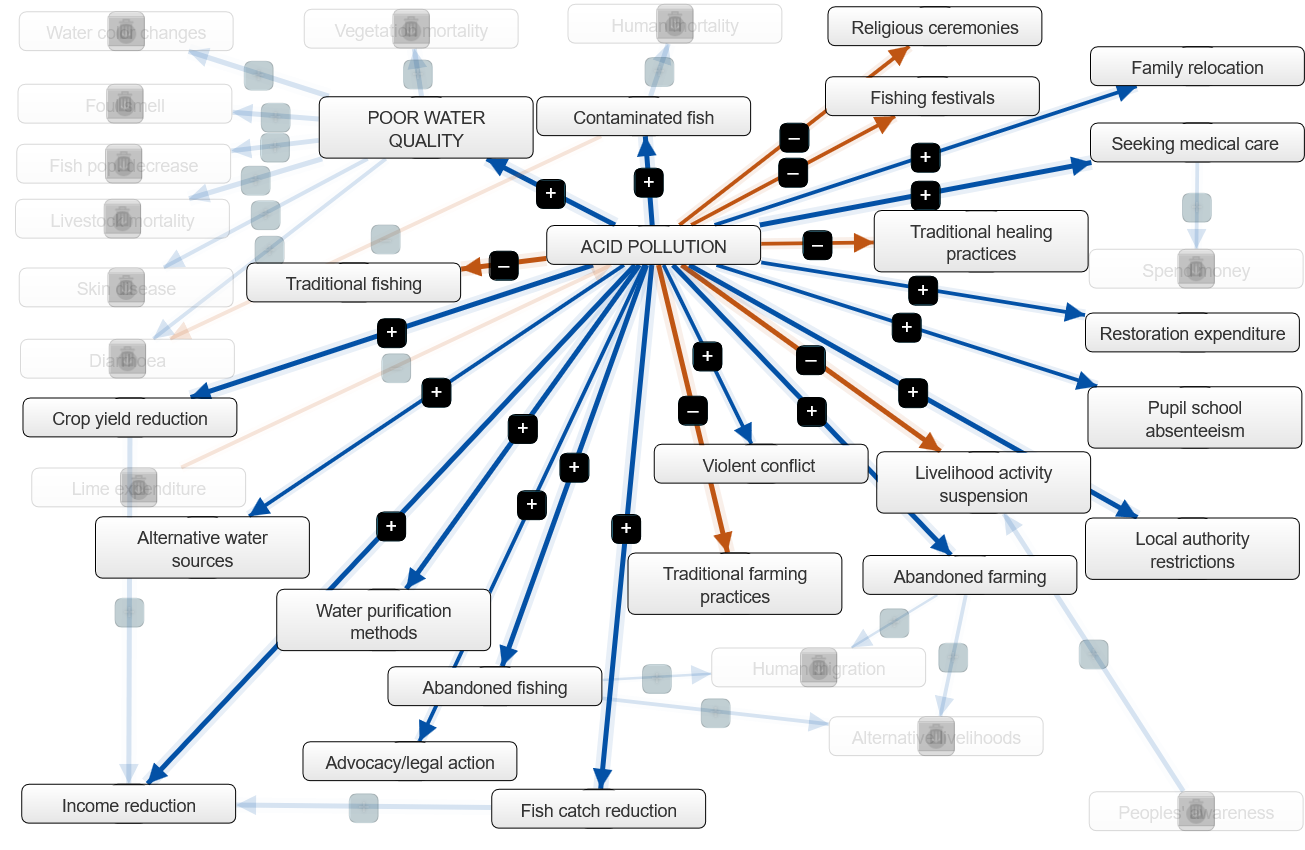


Figure SI 1: AMD as a variable is more extensive than any other variable in the FCM. Highlighted are the directly connected components and relationships with AMD. The blue arrows represent the positive causal effects between the components, while brown arrows depict the negative causal effects between the components. The thickness of lines reflects the strength of the relationships, and the arrowheads indicate the direction of the effects.


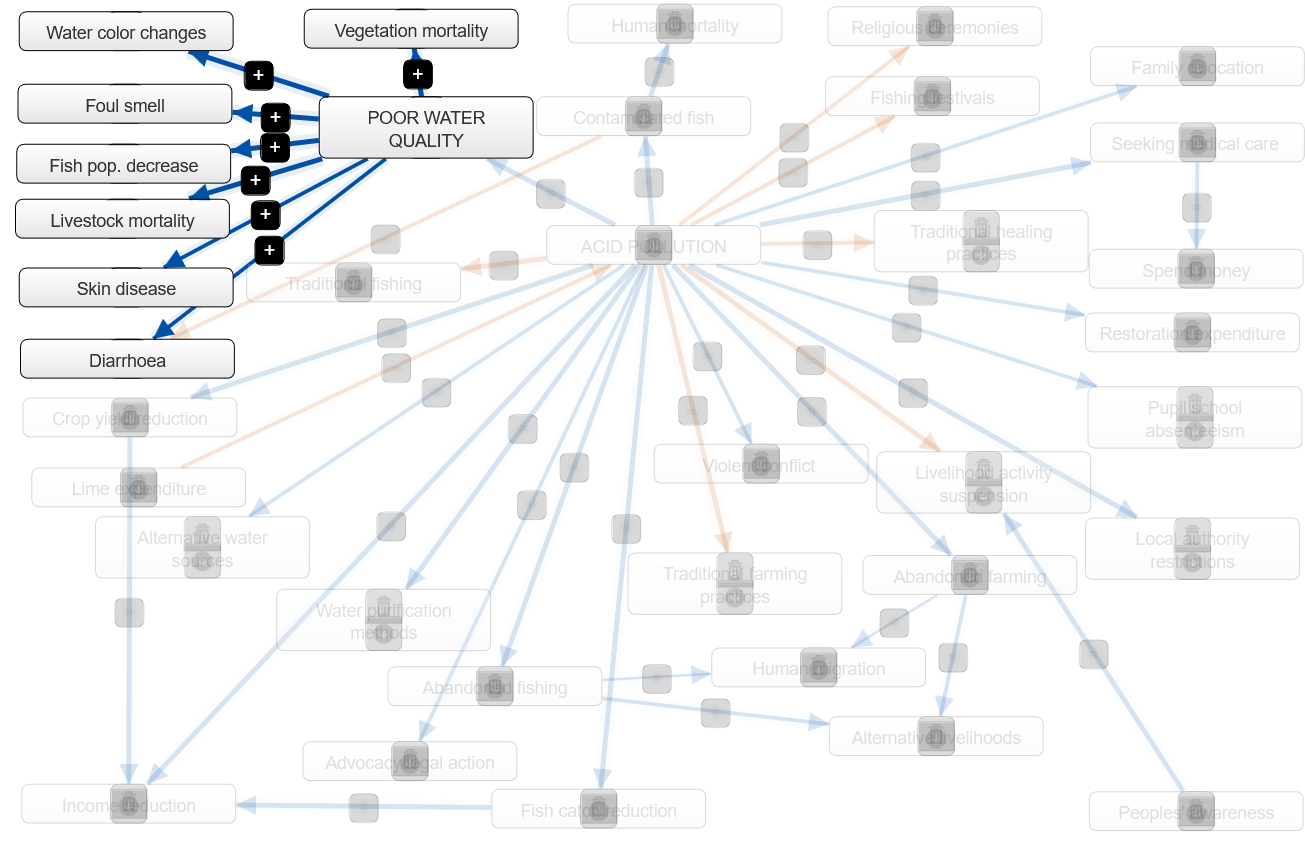


Figure SI 2: Poor water quality as the second most extensive variable in the FCM. Highlighted are the directly connected components and relationships with poor water quality. The blue arrows represent the positive causal effects between the components, while brown arrows depict the negative causal effects between the components. The thickness of lines reflects the strength of the relationships, and the arrowheads indicate the direction of the effects.

**Appendix A.1: Semi-structured questionnaire**

*Hello, I am _______________________. We invite you to be part of our research project activity which focuses on socio-economic impacts of acid pollution from the failed tailing dam at Sino Metals Leach Zambia. The survey takes approximately 40 minutes to complete. You must be 18 years or older to participate. You have the right to decline participation in any question, and to stop participation at any time. Your participation in this study is voluntary. If you choose not to participate or to withdraw from the study at any time, there will be no penalty. The information collected in this study will result in written reports, publications, and presentations, but your name will not be used. There are no foreseeable risks or discomforts to your participation. Your responses will be anonymous. If you have any questions or concerns about the research study, please contact ________________________.*

***Section 1: Basic information***

1. Serial No. ………………………………………………….
2. Name of participant (optional): ………………………………………………….
3. Location/township/village: ………………………………………………….
4. Age: ………………………………………………….
5. Gender (female / male): ………………………………………………….
6. Marital status: ………………………………………………….

[single/married/divorced/widowed/other]

1. Ethnic grouping (tribe): ………………………………………………….
2. Education level attained: ☐ No formal education ☐ Primary ☐ Secondary

☐ Tertiary

1. Family (household) size: ………………………………...……..members
2. Occupation: ☐ Fishing ☐ Farming ☐ Trading ☐ Formal

Employment ☐ Other (Specify)……………..

1. Organization: ………………………………………………….

***Section 2: Environmental and Health Impacts***

1. Have you noticed any changes in the water quality in the Mwambashi or Kafue River?

☐ Yes ☐ No

2. If yes, what changes have you observed? (Select all that apply)
☐ Change in water colour
☐ Foul (bad) smell
☐ Decrease in fish population
☐ Increased waterborne diseases
☐ Other (Specify) ____________

3. On a scale, 1-5 rank the changes (1 being small, 5 being large) ____________

4. Has anyone in your household experienced health issues linked to water pollution?

☐ Yes ☐ No

5. If yes, what health issues? (Select all that apply)
☐ Skin diseases
☐ Diarrhoea
☐ Respiratory issues
☐ Other (Specify) ____________

6. On a scale, 1-5 rank the severity of the health issues (1 being small, 5 being large) ____________

***Section 2: Economic effects***

7. Has acid pollution affected your main source of income? ☐ Yes ☐ No

8. If yes, how? (Select all that apply)
☐ Reduced fish catch
☐ Poor crop yields
☐ Increased cost of clean water
☐ Increased medical expenses
☐ Other (Specify) ____________

9. On a scale, 1-5 rank the loss (1 being small, 5 being large) ____________

10. Have you incurred additional costs due to water pollution? ☐ Yes ☐ No

11. If yes, what costs have increased? (Select all that apply)
☐ Medical bills
☐ Water treatment/purchase
☐ Loss of income
☐ Other (Specify) ____________

12. On a scale, 1-5 rank the increase in cost (1 being small, 5 being large) ____________
Key: **1**:<1%, **2**:1-5%, **3**:6-10%, **4**:11-15, **5**:>15%

***Section 2: Social and cultural effects***

13. Has pollution affected traditional fishing? ☐ Yes ☐ No

14. If yes, on a scale, 1-5 rank the degree of effects (1 being small, 5 being large) ________

15. Has pollution affected traditional farming practices? ☐ Yes ☐ No

16. If yes, on a scale, 1-5 rank the degree of effects (1 being small, 5 being large) ________

17. Has water pollution affected cultural or religious activities in the community? ☐ Yes ☐ No

18. If yes, which cultural /religious activities? (Select all that apply)
☐ Religious ceremonies
☐ Festivals
☐ Traditional healing practices
☐ Other (Specify) ____________

19. On a scale, 1-5 rank the degree of effects (1 being small, 5 being large) ____________

20. Have any traditional or local beliefs influenced how people respond to water pollution?

☐ Yes ☐ No

21. If yes, how? ____________________________________________________________

22. On a scale, 1-5 rank the degree of influence (1 being small, 5 being large) ___________

***Section 3: Opportunity costs***

23. What economic activities have you had to abandon or reduce due to pollution? (Select all that apply)
☐ Farming ☐ Fishing ☐ Trading ☐ Tourism ☐ Other (Specify) ______

24. On a scale, 1-5 rank the degree of abandonment/reduction (1 being small, 5 being large) ____________

25. How much income have you lost due to pollution since acid pollution occurred? (Estimate in local currency) ______

26. On a scale, 1-5 rank the degree of loss (1 being small, 5 being large) ____________

27. Have you had to switch to alternative livelihoods? ☐ Yes ☐ No

27.1. If yes, what are they? ______

28. How much time do you or your family members spend seeking medical care due to pollution-related illnesses?
☐ Less than 1 day/week ☐ 1-3 days/week ☐ More than 3 days/week

29. Have you had to spend money on medical treatment due to acid pollution? ☐ Yes ☐ No

30. If yes, what is the estimated cost of medical expenses per week? ______

31. On a scale, 1-5 rank the % of cost increase (1 being small, 5 being large) ____________

Key: **1**:<1%, **2**:1-5%, **3**:6-10%, **4**:11-15, **5**:>15%

32. Have children in your community missed school lessons due to pollution-related health issues?

☐ Yes ☐ No

33. On a scale, 1-5 rank the % of increase missed classes (1 being small, 5 being large) ____________

Key: **1**:<1%, **2**:1-5%, **3**:6-10%, **4**:11-15, **5**:>15%

34. Have any families relocated due to acid pollution? ☐ Yes ☐ No

35. Have community conflicts arisen due to pollution and its effects? ☐ Yes ☐ No

36. On a scale, 1-5 rank the severity of increase (1 being small, 5 being large) ___________

37. Have you or your community had to spend money to restore or adapt to environmental damage (e.g., buying clean water, soil rehabilitation, building alternative infrastructure)?

☐ Yes ☐ No

38. On a scale, 1-5 rank the % of cost increase (1 being small, 5 being large) ____________

Key: **1**:<1%, **2**:1-5%, **3**:6-10%, **4**:11-15, **5**:>15%

39. Have local authorities implemented policies that have restricted your access to land, water, or other resources due to pollution? ☐ Yes ☐ No

40. On a scale, 1-5 rank the level of restriction (1 being small, 5 being large) ____________

Key: **1**:<1%, **2**:1-5%, **3**:6-10%, **4**:11-15, **5**:>15%

41. Have you had to spend time or money engaging in advocacy or legal action related to pollution? ☐ Yes ☐ No

42. On a scale, 1-5 rank the % of cost increase (1 being small, 5 being large) ____________

Key: **1**:<1%, **2**:1-5%, **3**:6-10%, **4**:11-15, **5**:>15%

***Section 4: Coping strategies and recommendations (scenarios)***

43. What measures have you taken to cope with water pollution? (Select all that apply)
☐ Use of alternative water sources
☐ Water purification methods
☐ Change in livelihood activities
☐ Migration to other areas
☐ Other (Specify) ____________

44. On a scale, 1-5 rank the effectiveness of the measure (1 being small, 5 being large) ___

45. Which of additional **practical actions** should be taken by the stakeholders? (Select all that apply)
☐ Stricter environmental regulations
☐ Improved waste treatment facilities
☐ Community education and awareness
☐ Increased monitoring of industrial activities
☐ Other (Specify) ____________

46. On a scale, 1-5 rank the anticipated effectiveness of the measure (1 being small, 5 being large) ____________

47. What **technical solutions** do you think would help mitigate acid pollution in your community?

_________________________________________________________________________

48. What **policy recommendations** would you suggest to decision-makers to address the issues of acid pollutions?

_________________________________________________________________________

**End of Questionnaire
Thank you for your time and participation.**
